# Drivers and barriers of international migration of doctors to and from the United Kingdom: a scoping review

**DOI:** 10.1186/s12960-022-00789-y

**Published:** 2023-02-14

**Authors:** N. Brennan, N. Langdon, M. Bryce, L. Burns, N. Humphries, A. Knapton, T. Gale

**Affiliations:** 1 Collaboration for the Advancement of Medical Education Research, Peninsula Medical School, Faculty of Health, University of Plymouth, Plymouth, UK; 2 Strategic Modelling Analysis and Planning Limited (SMAP), Winchester, UK; 3 Graduate School of Healthcare Management, Royal College of Surgeons in Ireland, Dublin, Ireland

**Keywords:** Workforce planning, Workforce recruitment, Migration, Doctors

## Abstract

**Background:**

Many high-income countries are heavily dependent on internationally trained doctors to staff their healthcare workforce. Over one-third of doctors practising in the UK received their primary medical qualification abroad. Simultaneously, an average of around 2.1% of doctors leave the UK medical workforce annually to go overseas. The aim of this study was to identify the drivers and barriers of international migration of doctors to and from the UK.

**Methods:**

A scoping review was conducted. We searched EMBASE, MEDLINE, CINAHL, ERIC and BEI in January 2020 (updated October 2021). Grey literature and citation searching were also carried out. Empirical studies reporting on the drivers and barriers to the international migration of doctors to and from the UK published in the English language from 2009 to present were included. The drivers and barriers were coded in NVivo 12 building on an existing framework.

**Results:**

40 studies were included. 62% were quantitative, 18% were qualitative, 15% were mixed-methods and 5% were literature reviews. Migration into and out of the UK is determined by a variety of macro- (global and national factors), meso- (profession led factors) and micro-level (personal factors). Interestingly, many of the key drivers of migration to the UK were also factors driving migration *from* the UK, including: poor working conditions, employment opportunities, better training and development opportunities, better quality of life, desire for a life change and financial reasons. The barriers included stricter immigration policies, the registration process and short-term job contracts.

**Conclusions:**

Our research contributes to the literature by providing a comprehensive up-to-date review of the drivers and barriers of migration to and from the UK. The decision for a doctor to migrate is multi-layered and is a complex balance between push/pull at macro-/meso-/micro-levels. To sustain the UK’s supply of overseas doctors, it is vital that migration policies take account of the drivers of migration particularly working conditions and active recruitment while addressing any potential barriers. Immigration policies to address the impact of Brexit and the COVID-19 pandemic on the migration of doctors to and from the UK will be particularly important in the immediate future.

*Trial registration* PROSPERO CRD42020165748.

## Background

The global geographic mal-distribution of doctors has been an ongoing problem for decades [1] meaning access to health services everywhere in the world is disparate [2]. This mal-distribution of doctors is a particular challenge for the UK [3, 4]. According to the World Health Organisation, the UK has 2.8 doctors per 1000 population. This figure is well below the European Union (EU) average of 3.4/1000 with only four countries, Ireland, Slovenia, Romania, and Poland, having lower numbers of doctors per capita. Workforce challenges in the NHS in England are now a greater threat to the delivery of health services than funding challenges [5]. The UK’s attempts to address this issue have been comprised of both long-term and short-term strategies. These measures have included an expansion of undergraduate medical school places by 25%, improving working conditions for junior doctors [6, 7] and even a relaxation of immigration caps in 2018 to facilitate/enable greater international recruitment from outside the EU [8]. However despite these measures the BMA reported 10,582 vacancies in secondary care medical staff positions across the UK in 2022 [9] and the Royal College of Physicians census showed that only 52% of consultant vacances were filled in 2022 [10]. These figures illustrate the dire need for qualified medical specialists in the UK medical workforce.

Similar to New Zealand, Ireland, the USA and Canada, the United Kingdom (UK) is heavily dependent on internationally trained doctors to staff its healthcare system [11, 12]. Doctors with overseas qualifications are a core part of the UK medical workforce and 34.5% of licensed doctors in the UK received their primary medical qualification (PMQ) abroad [13]. At the same time, an average of 5% of doctors left the UK medical workforce each year from 2013 to 2019; data captured on their reasons for leaving demonstrated that approximately 2.1% are going overseas [12, 14]. The most popular places UK-trained doctors migrate to are other high-income English-speaking countries especially Australia and New Zealand [12].

There have been numerous theories proposed to understand migration over the years [15]. One of the most dominant theories has been the push–pull model which we will use in this paper as a framework to understand the migration of doctors to and from the UK. Push–pull models focus on disparities in conditions to explain migration patterns. Push–pull models in some of the early literature highlighted how migrants were pushed by low incomes in their countries and pulled by better opportunities in more prosperous areas [16, 17].

Central to the push–pull model of migration is the concept of ‘drivers’. Van Hear et al. [18] outlines how structural forces lead to both the inception of migration and the perpetuation of movement and these forces can be understood as the drivers of migration [19]. Similar to Van Hear et al. [18] we use the term driver to mean the variety of factors that may make up the external structural elements shaping the decision to migrate. Thus, drivers influence the broader context within which peoples hopes and wishes to migrate are formed and in which people ultimately make their decisions to migrate. Drivers may operate at different scales, levels of social structure, in different locations, at places of origin, transit and destination and over different timeframes [18].

There has been one systematic review conducted on the migration of doctors into the UK to date. This study was conducted by Davda in 2018 [20] and examined the migration motives of international dental graduates, compared with nurses and doctors in the United Kingdom. Based on the 31 studies included in the synthesis, the review identified common drivers including active recruitment, the desire to gain postgraduate training and financial gain; however, the extent to which each of these drivers influence healthcare professionals migration is different. This review only focused on qualitative studies, did not provide a detailed description of the migration drivers for the three different healthcare professionals included and is now out of date (searches completed in January 2017). On this basis we felt that an updated scoping review of the literature incorporating a broader range of study types focusing specifically on doctors was justified. In addition, we also felt it was important to identify the barriers to the migration of doctors to the UK also, as Davda focused on factors affecting inward migration to the UK only.

Thus the aim of this study was to identify the drivers and barriers of international migration of doctors to and from the UK. By summarising the most recent knowledge on this topic, the research will seek to inform policies on international recruitment globally, retention of the workforce and identify areas, where further research is needed for future workforce planning.

## Methods

### Type of review

Distinguishing between a scoping review and a systematic review of the literature can be difficult, as both share many essential characteristics namely, collecting, evaluating and presenting the available research evidence [21]. Originally, we planned to conduct a systematic review and developed and registered a protocol with PROSPERO (CRD42020165748). However, Munn et al. [21] argue that if a review is more interested in the identification of certain characteristics/concepts in papers or studies and in the mapping, reporting or discussion of these characteristics/concepts then a scoping review approach is the better choice. On reflection, as the aim of our review was to identify the key characteristics of the drivers and barriers relating to the migration of doctors, we have classified this review as a scoping review rather than a systematic review in this paper. The primary consequence of this change is that in line with scoping review methods [21], we did not undertake quality assessment of the included papers. This review was informed by Arksey and O’Malley’s methodological framework for scoping reviews [22].

### Review protocol

A review protocol was developed by the research team and was registered with PROPSERO (a prospective register of systematic reviews) to help avoid unplanned duplication and to enable comparison of reported review methods with what was planned in the protocol [23]. The PROSPERO reference number is CRD42020165748.

### Search strategy

The search strategy was designed, piloted and carried out by an experienced information specialist (LB). The search strategy aimed for comprehensiveness through the extent and range of searching. We searched both medical and other health professions databases (EMBASE, MEDLINE, CINAHL) as well as educational databases (e.g. ERIC, BEI). The searches were carried out on the 23rd of January 2020 and were subsequently updated on the 13th of October 2021. We searched for relevant items from 2009 to 2021 in order to build a picture of the current migration drivers and barriers. The databases were searched with free text keywords and controlled vocabulary where appropriate using terms such as ‘doctors’ OR ‘physician’ AND ‘migration’ OR ‘emigration’ or ‘brain drain’ OR ‘working overseas’ OR ‘come to the UK’ OR ‘overseas trained’ OR ‘internationally trained’. See Appendix A for full search histories.

Grey literature searching was also carried out. The grey literature databases HMIC and British Library EThOS were searched on the 13th of May 2020. The websites of key organisations were searched for relevant publications on the 30th of May 2020. Key organisations that conduct research on migration were included i.e. the WHO, the ONS, Euro Stat and the OECD. We also searched the websites of the regulators of the countries UK doctors typically migrate to including the Medical Council of Ireland (MCI), GMC, Australian Medical Council, New Zealand Medical Council, Medical Council of Canada and the American Medical Association.

We searched the bibliographies of included papers. The abstracts for any relevant papers were sought and then the inclusion criteria were applied.

### Study selection

In order to select studies relevant to our research questions we applied the following inclusion criteria:

- Topic of interest—the international migration of doctors.
- Aspect of topic—drivers and barriers of migration to and from the UK
- Countries of interest—the UK. Any study that reported the drivers and barriers of migration to and from the UK
- Type of participants—all studies about doctors.
- Study design—all articles that reported empirical research. Literature reviews were also included if they were systematic reviews of the literature or reported systematic search methods.
- Language—studies published in English language.
- Date—2009 to present. This was a sufficient timeframe to access recent relevant literature.
- Outcome measures—all outcome measures.

The potential relevance of all titles and abstracts was assessed using Rayyan QCRI (systematic review software) by three reviewers independently (NL, NB and LB). All articles were double screened and any discrepancies were discussed until agreement was reached.

### Data extraction

The papers of all eligible studies were obtained and read in full. A standardised data extraction review form was piloted and utilised. The extraction was carried out in Microsoft Excel by two researchers (NL and NB). A random sample of 10% of all articles were data extracted independently by both researchers and then compared for consistency.

As scoping reviews do not aim to produce a critically appraised and synthesised result/answer to a particular question, and rather aim to provide an overview or map of the evidence, an assessment of methodological limitations or risk of bias of the evidence was not performed [21].

### Data synthesis

Data extracted on drivers and barriers was then exported into NVivo 12 (QSR). NVivo is a computerised indexing system for coding and analysing qualitative data. In order to consistently categorise the drivers and barriers we decided to develop a coding framework. As a coding framework had already been developed by Young [24] and successfully used in the Davda [20] systematic review we decided to use the same framework as a starting point for coding our data. Young’s model categorises the factors attracting health professionals to the UK into three broad categories including macro-level (global and national factors), meso-level (profession led factors) and micro-level (personal factors) drivers of migration [24]. Additional codes identified in our data were added to Young’s framework. See Appendix B for the full coding framework. We also developed another arm to the coding framework to capture the barriers. A 10% sample of the articles was coded by two reviewers (NL and NB) to compare for consistency. One reviewer then coded the remaining papers (NL).

In the results section we present the top three macro-, meso- and micro-level drivers and barriers. A driver or barrier was identified as being in the top three based on the number of articles that had identified the driver or barrier. If a driver or barrier was identified more than once in a paper we only counted it once.

The included papers were classified according to the main country or region that the data collection related to e.g. the UK, Ireland, Africa/Asia. However, this did not mean that the paper was solely about this country/or region. For example sometimes a paper may have been classified as being about Europe but the paper may have contained a driver or barrier relating specifically to the UK.

## Results

### Literature identified

**Fig. 1:**
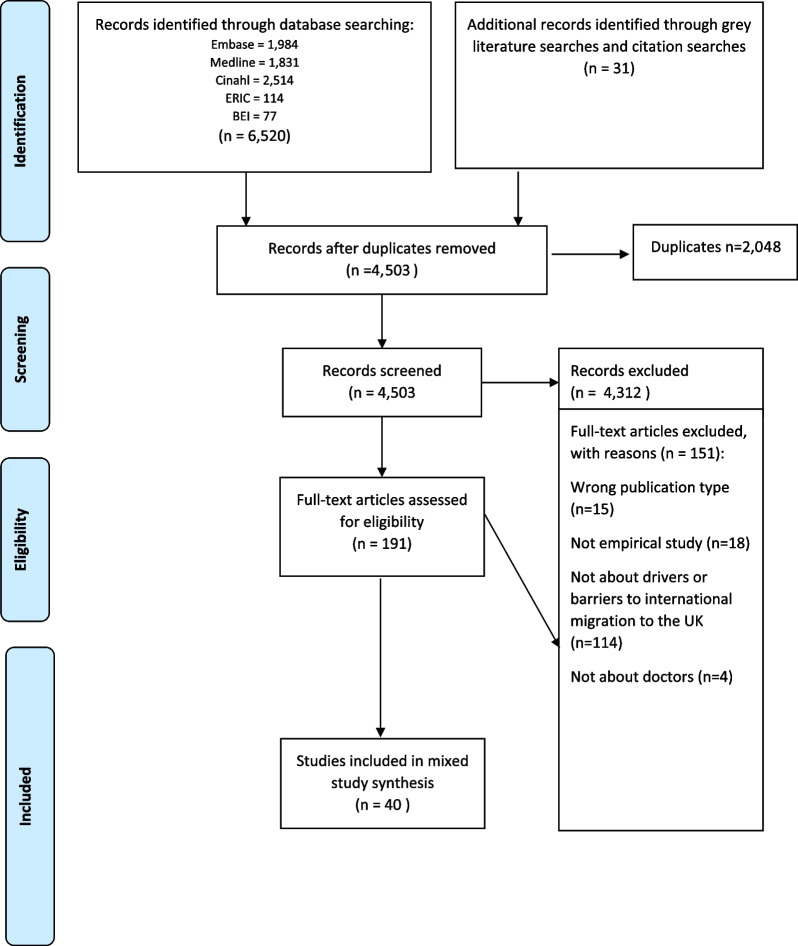
PRISMA Flow Diagram

The search identified 4512 potentially relevant articles and, after the inclusion criteria were applied, 40 articles were included in the review (Fig. 1). The characteristics of the 40 included studies are listed in Appendix C.

### Characteristics of included studies

62% of studies were quantitative, 18% were qualitative, 15% were mixed methods and 5% were literature reviews. The main focus of 60% of the studies was migration to and from the UK, 15% were about European countries, 10% were about African or Asian countries, 10% were about Ireland and 5% were about Australia/New Zealand.

### What are the drivers of the migration of doctors to and from the UK?

The drivers we identified are summarised in Table 1. We found that many of the reasons associated with attracting doctors to the UK, also constituted push factors for UK doctors to decide to leave the UK and practise abroad.

**Table 1:** Drivers and barriers to the migration of doctors to and from the UK

Macro-level drivers	
DRIVERS TO THE UK	DRIVERS FROM THE UK
1.5* Employment opportunities [20, 25–30]	1.16* Poor working conditions [31–38]
1.1 Active recruitment [20, 29, 39–43]	1.6 Attractive working conditions elsewhere [32, 34–38, 44, 46]
1.16 Poor working conditions [20, 37, 39, 42, 46, 47]	1.5 Employment opportunities [32, 34, 37, 44]
Meso-level drivers	
2.1 Better training and development opportunities [20, 26, 27, 29, 39, 42, 46, 48–52]	2.14 Pushed/desire to leave the NHS [31, 33, 34, 36, 38, 44]
2.10 Desire to experience working in a different environment [20, 39, 42, 46, 48, 50]	2.1 Better training and development opportunities [32, 36, 38, 53]
2.19 Opportunities to gain clinical experience through short-term employment [20, 39, 42, 46, 48, 50]	2.17 Undervalued professionally [32, 35, 54]
Micro-level drivers	
3.17 Financial gain for self (and/or family) [20, 26, 27, 37, 39, 42, 47, 48, 51, 55–57]	3.1 Better quality of life [32–36, 38, 44]
3.2: Desire for life change [20, 37, 39, 42, 46, 50]	3.4 Family reasons [33–35, 37, 46]
3.1: Better quality of life [39, 42, 46, 48, 51]	3.2 Desire for a life change [32–34, 36–38, 46]

*The numbers correspond to the codes in the coding framework in Appendix C

### What are the barriers to doctors coming to work in the UK and to leave the UK?

The main barriers impeding doctor migration to and from the UK are presented in Table 2.

**Table 2:** Barriers to the migration of doctors to and from the UK

Macro-level barriers	
Coding descriptor	References
4.21*Stricter immigration policies	[35, 40, 42, 58, 59]
4.26 Process of gaining registration	[27, 48, 60, 61]
4.1 Healthcare system difficult to enter or differences in healthcare system	[27, 42, 48]
Meso-level Barriers	
5.14 Short-term job contracts	[35, 62]
5.3 Limited training opportunities	[32, 42]
5.16 Negative experience of induction scheme	[27]
Micro-level barriers	
6.8 Concerns about a new working environment	[46, 48]
6.19 Lack of support	[48, 62]
6.22 Language difficulties	[27, 46]

*The numbers correspond to the codes in the coding framework in Appendix C

## Discussion

### Statement of principal findings and comparison with existing literature

This comprehensive review of the literature set out to identify the drivers and barriers of international migration of doctors to and from the UK. By summarising the most recent knowledge on this topic, the research will inform policies on international recruitment, retention of the workforce and identify future workforce planning research gaps. We identified a variety of push and pull factors at the macro- (global and national), meso- (professional) and micro- (personal) levels. Interestingly, many of the key drivers of migration to the UK were also factors driving migration from the UK to other countries and are thus relevant for all high-income countries. These included: poor working conditions, employment opportunities, better training and development opportunities, better quality of life, desire for a life change and personal financial gain. The barriers to the migration of doctors to and from the UK included stricter immigration policies, the process of gaining registration, short-term job contracts, limited training opportunities, concerns about a new working environment and lack of support.

The findings of the review highlight how the decision for a doctor to migrate is multi-layered and is a complex balance between push and pull factors at macro-/meso-/micro-levels. The decision to migrate is also relative to a doctors’ own values and experiences, reflecting individual priorities. Furthermore, as Franco et al. point out “not all workers will have the same mix of motives and goals, and the relative importance of particular values and work goals will change over time and situations” [63: 1258]. While the push–pull model of migration has been criticised for being simplistic [64], Van Hear highlights the strengths of the simple notion of push–pull, “with its intuitive and empirically grounded idea that structural forces shape migration processes” [18: 928]. The challenge for researchers is to establish when and why some drivers are more important than others, which combinations are more potent than others, and which are more susceptible to change through external intervention. This complexity poses a significant challenge to policy-makers in terms of planning and ensuring a supply of doctors for the medical workforce. In order to sustain the UK’s supply of overseas doctors, it is vital that UK migration policy relating to doctor migration take account of the drivers of migration while addressing any potential barriers.

Policy-makers are most likely to have an impact on the macro- and meso-level factors we have identified in the review as the micro-level factors i.e. financial gain, better quality of life and family reasons, relate to an individual’s personal values and circumstances. The most important macro-level driver of migration to the UK was employment opportunities. Due to the UKs current shortage of doctors it is likely that there will continue to be employment opportunities particularly in some specialities e.g. general practice, psychiatry and emergency medicine. Thus, policymakers should focus on ensuring the employment opportunities are advertised globally as well as focusing on ethical active recruitment for jobs (another of the macro-level drivers).

The main macro-level factor policy-makers can have a big impact on and should prioritise is working conditions in the NHS. Policy-makers are well aware of the need to improve working conditions in the NHS and this has been addressed in the 5 year plan [65, 66] and other initiatives focusing in particular on NHS staff health and well-being [67, 68]. Humphries also found in her study of the migration of doctors from Ireland to Australia that working conditions are a particularly important factor in migration decisions and whatever health system gets this aspect right will be successful in attracting and retaining doctors [49, 69]. The other macro-level factor policy-makers can target is training and development opportunities and putting policies in place to ensure the high standards of training the UK is renowned for are available for overseas trained doctors who are not yet qualified to join the specialist or GP registers.

The opposite side of the push–pull model driving migration of overseas doctors into the UK, are barriers to migration. These factors may hinder, or make difficult the ability to migrate, or they may be factors that dissuade doctors from migration, for example, positive improvements to salary or working conditions in one’s home country. The literature contained few references to the barriers to migration, prioritising instead the push/pull factors driving doctors to either immigrate into, or emigrate out of, the UK. The likely reason for this is that methodologically it is much more difficult to ascertain changed intentions to migrate, and what may have changed or obstructed a nascent decision to migrate [18, 64].

The review highlighted that stricter immigration policies and the process of registration were significant barriers. These barriers are particularly relevant for EU doctors in the BREXIT era. The fact that EU doctors qualifications are now not automatically recognised in the UK means the number of doctors migrating to the UK from the EU could decrease [70, 71]. Recent projections are that Brexit has resulted in 4,285 fewer European doctors coming to the UK [70]. Thus, policy makers will need to consider how they can make up this potential shortfall in the supply of doctors from the EU.

The findings of our review corroborate the findings of the Davda [20] review which identified active recruitment, postgraduate training and financial gain as strong common macro, meso and micro drivers that perpetuate migration into the UK. It is not surprising that the findings were similar, as the Davda review was an included paper in our review and there was also some overlap between included studies in the two reviews. Davda included 31 studies in their review, five of which were also in ours. However, despite this overlap there was still quite a number of different studies involved in our review (*n* = 34) with some focusing on the recent impacts of BREXIT and the COVID-19 pandemic on migration patterns. The fact that the findings were similar adds more weight to the drivers identified in both reviews. It also implies that these drivers have not changed significantly over time.

Studies on Ireland [72, 73] and other higher income countries in Europe [39] also reported poor working conditions, employment opportunities, better training and development opportunities, financial gain, better quality of life and family reasons as being important drivers of emigration. This begs the question: how can high income countries maintain a competitive advantage in this area? The COVID-19 pandemic has served as a reminder of how important the medical workforce is. Post COVID-19 health systems everywhere will be looking to strengthen their medical workforce and as this will influence expectations, future plans and migration decisions [52] it will also increase international competition.

### Strengths and limitations

This is a comprehensive review of the literature. The search strategy was designed by an experienced information specialist and a variety of medical and educational databases were searched. All relevant papers identified were double-screened and data extraction consistency checks were carried out for 10% of papers. The review team were multidisciplinary including social scientists and clinicians. We included studies that were not primarily about the UK that contained drivers and barriers and we also included all study designs so a broader literature was drawn upon.

As with all research studies, there are also some limitations. A limitation of the study is that we only identified and classified the drivers and barriers from the literature and so if a driver was identified many times in a study we only reported it once. We did not carry out a quality assessment so cannot comment on the strength of the evidence underpinning each driver and barrier identified. As with all literature reviews the results are dependent on the quality of the existing literature and due to very limited literature on the barriers we were not able to address this question sufficiently. Resource constraints meant we only looked at literature since 2009 and published in the English language.

## Conclusion

Our research contributes to the literature by providing an up-to-date review of the drivers and barriers of migration to and from the UK. The review found that the decision for a doctor to migrate is multi-layered and is a complex balance between push/pull at macro-/meso-/micro-levels. This complexity poses a significant challenge to policymakers in planning and ensuring a supply of doctors for high income countries. To sustain the UK’s supply of overseas doctors, it is vital that migration policy tools are developed that recognise and support the drivers of migration particularly working conditions and active recruitment while addressing any potential barriers particularly immigration policies and the process for gaining registration. Immigration policies to address the impact of Brexit and the COVID-19 pandemic on the migration of doctors to and from the UK will be particularly important in the immediate future.

## Appendix A

### Systematic review search histories

**Search Histories 23.01.2020.**

**Table:** 

Database	Results
Ovid Embase	1631
Ovid MEDLINE	1533
CINAHL via EBSCOhost	2085
ERIC via EBSCOhost	109
BEI via EBSCOhost	62
Total before dedup	5423

**Table:** 

Embase < 1974 to 2020 January 23 >		
Search history sorted by search number ascending		
#	Searches	Results
1	exp physician/	720,194
2	("doctor*" or physician or "general practitioner*" or "GP*" or "G.P*").tw	667,950
3	"physician*".tw	530,375
4	1 or 2 or 3	1,353,626
5	foreign medical graduate/ or foreign physician/	277
6	((emigrat* or migrat* or immigrat*) adj3 (doctor* or physician* or "general practitioner*" or "GP*" or "G.P*")).tw	775
7	((work* or practi?e) adj3 (overseas or abroad or "another country")).tw	940
8	"brain drain".tw	620
9	((work or practise) adj3 (UK or Britain or "U.K." or EU or "European Union" or "E.U." or Ireland or Canada or Australia or Zealand or USA or "U.S.A" or America)).tw	1569
10	((leave or come to) adj3 (UK or Britain or "U.K." or EU or "European Union" or "E.U." or Ireland or Canada or Australia or Zealand or USA or "U.S.A" or America)).tw	359
11	((trained or qualified or graduated) adj3 (overseas or abroad or international* or "another country")).tw	511
12	international medical graduate*.tw	943
13	((doctor* or physician* or "general practitioner*" or "GP*" or "G.P*") adj2 (mobility or movement)).tw	332
14	5 or 6 or 7 or 8 or 9 or 10 or 11 or 12 or 13	5966
15	4 and 14	2662
16	limit 15 to year = "2009—2020"	1705
17	limit 16 to English language	1631

**Table:** 

Ovid MEDLINE(R) and Epub Ahead of Print, In-Process & Other Non-Indexed Citations, Daily and Versions(R) < 1946 to January 23, 2020 >		
Search history sorted by search number ascending		
#	Searches	Results
1	exp Physicians/	136,160
2	("doctor*" or "general practitioner*" or "GP*" or "G.P*").tw	323,888
3	"physician*".tw	377,649
4	1 or 2 or 3	734,837
5	foreign medical graduates/	3443
6	((emigrat* or migrat* or immigrat*) adj3 (doctor* or physician* or "general practitioner*" or "GP*" or "G.P*")).tw	684
7	((work* or practi?e) adj3 (overseas or abroad or "another country")).tw	826
8	"brain drain".tw	612
9	((work or practise) adj3 (UK or Britain or "U.K." or EU or "European Union" or "E.U." or Ireland or Canada or Australia or Zealand or USA or "U.S.A" or America)).tw	1094
10	((leave or come to) adj3 (UK or Britain or "U.K." or EU or "European Union" or "E.U." or Ireland or Canada or Australia or Zealand or USA or "U.S.A" or America)).tw	277
11	((trained or qualified or graduated) adj3 (overseas or abroad or international* or "another country")).tw	391
12	international medical graduate*.tw	809
13	((doctor* or physician* or "general practitioner*" or "GP*" or "G.P*") adj2 (mobility or movement)).tw	289
14	5 or 6 or 7 or 8 or 9 or 10 or 11 or 12 or 13	7462
15	4 and 14	4973
16	limit 15 to yr = "2009—2020"	1662
17	limit 16 to english language	1533

**Table:** 

#	**Query—Database—CINAHL Plus with Full Text**	Results
1	(MH "Physicians + ")	103,131
2	TI ( "doctor*" or "general practitioner*" or "GP*" or "G.P*" or "medical personnel") OR AB ( "doctor*" or "general practitioner*" or "GP*" or "G.P*" or "medical personnel")	85,840
3	TI physician* OR AB physician*	128,694
4	S1 OR S2 OR S3	269,064
5	(MH "Foreign Medical Graduates")	795
6	TI ( (emigrat* or migrat* or immigrat*) N3 (doctor* or physician* or "general practitioner*" or "GP*" or "G.P*")) OR AB ( (emigrat* or migrat* or immigrat*) N3 (doctor* or physician* or "general practitioner*" or "GP*" or "G.P*")	204
7	TI ( (work* or practi?e) N3 (overseas or abroad or "another country")) OR AB ( (work* or practi?e) N3 (overseas or abroad or "another country"))	564
8	TI ( (work* or practise) N3 (UK or Britain or "U.K." or EU or "European Union" or "E.U." or Ireland or Canada or Australia or Zealand or USA or "U.S.A" or America)) OR AB ( (work* or practise) N3 (UK or Britain or "U.K." or EU or "European Union" or "E.U." or Ireland or Canada or Australia or Zealand or USA or "U.S.A" or America))	10,406
9	TI ( (leave or come) N3 (UK or Britain or "U.K." or EU or "European Union" or "E.U." or Ireland or Canada or Australia or Zealand or USA or "U.S.A" or America)) OR AB ( (leave or come) N3 (UK or Britain or "U.K." or EU or "European Union" or "E.U." or Ireland or Canada or Australia or Zealand or USA or "U.S.A" or America))	469
10	TI ( (trained or qualified or graduated) N3 (overseas or abroad or international* or "another country")) OR AB ( (trained or qualified or graduated) N3 (overseas or abroad or international* or "another country"))	296
11	TI ("international medical graduate*" or "brain drain") OR AB ("international medical graduate*" or "brain drain")	598
12	TI ((doctor* or physician* or "general practitioner*" or "GP*" or "G.P*") N2 (mobility or movement)) OR AB ((doctor* or physician* or "general practitioner*" or "GP*" or "G.P*") N2 (mobility or movement))	118
13	S5 OR S6 OR S7 OR S8 OR S9 OR S10 OR S11 OR S12	12,831
14	S4 AND S13	3119
15	S4 AND S13	3119
16	S4 AND S13	2085

**Table:** 

#	**Query—Database—British Education Index; ERIC**	Results
1	(MH "Physicians + ")	15
2	TI ( "doctor*" or "general practitioner*" or "GP*" or "G.P*" or "medical personnel") OR AB ( "doctor*" or "general practitioner*" or "GP*" or "G.P*" or "medical personnel")	22,431
3	TI physician* OR AB physician*	6,182
4	S1 OR S2 OR S3	28,133
5	(MH "Foreign Medical Graduates")	1218
6	TI ( (emigrat* or migrat* or immigrat*) N3 (doctor* or physician* or "general practitioner*" or "GP*" or "G.P*")) OR AB ( (emigrat* or migrat* or immigrat*) N3 (doctor* or physician* or "general practitioner*" or "GP*" or "G.P*")	35
7	TI ( (work* or practi?e) N3 (overseas or abroad or "another country")) OR AB ( (work* or practi?e) N3 (overseas or abroad or "another country"))	449
8	TI ( (work* or practise) N3 (UK or Britain or "U.K." or EU or "European Union" or "E.U." or Ireland or Canada or Australia or Zealand or USA or "U.S.A" or America)) OR AB ( (work* or practise) N3 (UK or Britain or "U.K." or EU or "European Union" or "E.U." or Ireland or Canada or Australia or Zealand or USA or "U.S.A" or America))	6114
9	TI ( (leave or come) N3 (UK or Britain or "U.K." or EU or "European Union" or "E.U." or Ireland or Canada or Australia or Zealand or USA or "U.S.A" or America)) OR AB ( (leave or come) N3 (UK or Britain or "U.K." or EU or "European Union" or "E.U." or Ireland or Canada or Australia or Zealand or USA or "U.S.A" or America))	507
10	TI ( (trained or qualified or graduated) N3 (overseas or abroad or international* or "another country")) OR AB ( (trained or qualified or graduated) N3 (overseas or abroad or international* or "another country"))	131
11	TI ("international medical graduate*" or "brain drain") OR AB ("international medical graduate*" or "brain drain")	407
12	TI ((doctor* or physician* or "general practitioner*" or "GP*" or "G.P*") N2 (mobility or movement)) OR AB ((doctor* or physician* or "general practitioner*" or "GP*" or "G.P*") N2 (mobility or movement))	37
13	S5 OR S6 OR S7 OR S8 OR S9 OR S10 OR S11 OR S12	7548
14	S4 AND S13	310
15	S4 AND S13	310
16	S4 AND S13 174 = 109 in ERIC and 65 in BEI	174

**Table:** 

#	Database	Search term	Results
1	HMIC	exp "MEDICAL STAFF"/	22,072
2	HMIC	"doctor*" OR physician OR "general practitioner*" OR "GP*" OR "G.P*"	35,935
3	HMIC	(1 OR 2)	42,114
4	HMIC	exp "LABOUR MOBILITY"/	61
5	HMIC	"LABOUR MIGRATION"/	87
6	HMIC	(emigrat* OR migrat* OR immigrat*) ADJ3 (doctor* OR physician* OR "general practitioner*" OR "GP*" OR "G.P*")	50
7	HMIC	((work* OR practi?e) ADJ3 (overseas OR abroad OR "another country")).ti,ab	166
8	HMIC	("brain drain").ti,ab	34
9	HMIC	(work OR practise) ADJ3 (UK OR Britain OR "U.K." OR EU OR "European Union" OR "E.U." OR Ireland OR Canada OR Australia OR Zealand OR USA OR "U.S.A" OR America)	271
10	HMIC	(leave OR come to) ADJ3 (UK OR Britain OR "U.K." OR EU OR "European Union" OR "E.U." OR Ireland OR Canada OR Australia OR Zealand OR USA OR "U.S.A" OR America)	80
11	HMIC	((trained OR qualified OR graduated) ADJ3 (overseas OR abroad OR international* OR "another country")).ti,ab	80
12	HMIC	(doctor* OR physician* OR "general practitioner*" OR "GP*" OR "G.P*") ADJ2 (mobility OR movement)	28
13	HMIC	(4 OR 5 OR 6 OR 7 OR 8 OR 9 OR 10 OR 11 OR 12)	766
14	HMIC	(3 AND 13)	247
15	HMIC	14 [DT 2009–2020]	**82 (27 after de-dup)**

**Table:** 

BL EThOS: Doctor (title) AND migration (title) AND UK (abstract) = 1 result
The migration of medical doctors from Poland to the United Kingdom following the expansion of the European Union in May 2004 https://discovery.ucl.ac.uk/id/eprint/1302279/ (not available)
Doctors (title) AND migration (title) Doctors (title) AND “brain drain” (abstract) Doctors (title) AND immigration (title) Doctors (title) AND emmigration (title) Doctors (title) AND migrate (title) Doctors (title) AND abroad (title) Doctors (title) AND Britain (title) Doctors (title) AND United Kingdom (title) General Practitioners (title) AND United Kingdom (title) General Practitioners (title) AND Britain (title) Doctors (title) AND abroad (title) General Practitioners (title) AND migration (title) General Practitioners (title) AND migrate (title) General Practitioners (title) AND immigration (title) General Practitioners (title) AND emmigration (title) General Practitioners (title) AND mobility (title) = 0 results

Search Histories 13.10.21.

**Table:** 

Database	Results
Ovid Embase	353
Ovid MEDLINE	298
CINAHL via EBSCOhost	429
ERIC via EBSCOhost	5
BEI via EBSCOhost	15
Total before dedup	1088
After dedup	782

**Embase < 1974 to 2021 October 12 >**

1exp physician/830272.

2("doctor*" or physician or "general practitioner*" or "GP*" or "G.P*").tw.743173.

3"physician*".tw.589350.

41 or 2 or 31,530,923.

5foreign medical graduate/ or foreign physician/390.

6((emigrat* or migrat* or immigrat*) adj3 (doctor* or physician* or "general practitioner*" or "GP*" or "G.P*")).tw.846.

7((work* or practi?e) adj3 (overseas or abroad or "another country")).tw.1034.

8"brain drain".tw.674.

9((work or practise) adj3 (UK or Britain or "U.K." or EU or "European Union" or "E.U." or Ireland or Canada or Australia or Zealand or USA or "U.S.A" or America)).tw.1763.

10((leave or come to) adj3 (UK or Britain or "U.K." or EU or "European Union" or "E.U." or Ireland or Canada or Australia or Zealand or USA or "U.S.A" or America)).tw.409.

11((trained or qualified or graduated) adj3 (overseas or abroad or international* or "another country")).tw.593.

12international medical graduate*.tw.1097.

13((doctor* or physician* or "general practitioner*" or "GP*" or "G.P*") adj2 (mobility or movement)).tw.396.

145 or 6 or 7 or 8 or 9 or 10 or 11 or 12 or 136,767.

154 and 143,045.

16limit 15 to yr = "2020—2021"363.

17limit 16 to english language**353.**

**Ovid MEDLINE(R) and Epub Ahead of Print, In-Process, In-Data-Review & Other Non-Indexed Citations, Daily and Versions(R) < 1946 to October 12, 2021 >**

1exp Physicians/156743.

2("doctor*" or "general practitioner*" or "GP*" or "G.P*").tw.360787.

3"physician*".tw.415660.

41 or 2 or 3,817,995.

5foreign medical graduates/3553.

6((emigrat* or migrat* or immigrat*) adj3 (doctor* or physician* or "general practitioner*" or "GP*" or "G.P*")).tw.752.

7((work* or practi?e) adj3 (overseas or abroad or "another country")).tw.893.

8"brain drain".tw.660.

9((work or practise) adj3 (UK or Britain or "U.K." or EU or "European Union" or "E.U." or Ireland or Canada or Australia or Zealand or USA or "U.S.A" or America)).tw.1243.

10((leave or come to) adj3 (UK or Britain or "U.K." or EU or "European Union" or "E.U." or Ireland or Canada or Australia or Zealand or USA or "U.S.A" or America)).tw.320.

11((trained or qualified or graduated) adj3 (overseas or abroad or international* or "another country")).tw.454.

12international medical graduate*.tw.942.

13((doctor* or physician* or "general practitioner*" or "GP*" or "G.P*") adj2 (mobility or movement)).tw.338.

145 or 6 or 7 or 8 or 9 or 10 or 11 or 12 or 138,106.

154 and 145,279.

16limit 15 to yr = "2020—current"303.

17limit 16 to english language**298.**

**CINAHL; ERIC; BEI.**

**Table:** 

#	Query	Results
S1	(MH "Physicians + ")	123,630
S2	TI ( "doctor*" or "general practitioner*" or "GP*" or "G.P*" or "medical personnel") OR AB ( "doctor*" or "general practitioner*" or "GP*" or "G.P*" or "medical personnel")	131,231
S3	TI physician* OR AB physician*	163,335
S4	S1 OR S2 OR S3	357,698
S5	(MH "Foreign Medical Graduates")	905
S6	TI ( (emigrat* or migrat* or immigrat*) N3 (doctor* or physician* or "general practitioner*" or "GP*" or "G.P*")) OR AB ( (emigrat* or migrat* or immigrat*) N3 (doctor* or physician* or "general practitioner*" or "GP*" or "G.P*")	295
S7	TI ( (work* or practi?e) N3 (overseas or abroad or "another country")) OR AB ( (work* or practi?e) N3 (overseas or abroad or "another country"))	1,163
S8	TI ( (work* or practise) N3 (UK or Britain or "U.K." or EU or "European Union" or "E.U." or Ireland or Canada or Australia or Zealand or USA or "U.S.A" or America)) OR AB ( (work* or practise) N3 (UK or Britain or "U.K." or EU or "European Union" or "E.U." or Ireland or Canada or Australia or Zealand or USA or "U.S.A" or America))	22,672
S9	TI ( (leave or come) N3 (UK or Britain or "U.K." or EU or "European Union" or "E.U.")) OR AB ( (leave or come) N3 (UK or Britain or "U.K." or EU or "European Union" or "E.U."))	313
S10	TI ( (trained or qualified or graduated) N3 (overseas or abroad or international* or "another country")) OR AB ( (trained or qualified or graduated) N3 (overseas or abroad or international* or "another country"))	496
S11	TI ("international medical graduate*" or "brain drain") OR AB ("international medical graduate*" or "brain drain")	1,143
S12	TI ((doctor* or physician* or "general practitioner*" or "GP*" or "G.P*") N2 (mobility or movement)) OR AB ((doctor* or physician* or "general practitioner*" or "GP*" or "G.P*") N2 (mobility or movement))	201
S13	S5 OR S6 OR S7 OR S8 OR S9 OR S10 OR S11 OR S12	26,328
S14	S4 AND S13	4,549
S16	S4 AND S13 Limiters—Publication Date: 20,200,101–20,211,231	**449**

**CINAHL: 429; BEI: 15 ERIC: 5**

## Appendix B

### Coding framework for systematic review

**Table:** 

Level of analysis	Themes and descriptions	Codes	Label
1. Macro-level drivers	Health system factors	1.1	Active recruitment
		1.2	Passive recruitment
		1.3	Good healthcare infrastructure
		1.4	Workforce demand
		1.5	Employment opportunities
		1.6	Attractive working conditions
		1.7	Safety and security of NHS
		1.8	Support offered for relocation induction
		1.9	Overproduction of nurses and doctors
		1.10	Low unemployment
		1.11	Unemployment
		1.12	Underemployment
		1.13	Poor healthcare infrastructure
		1.14	Poor job opportunities
		1.15	Poor salaries
		1.16	Poor working conditions
		1.17	Lack of support
		1.18	Ease of assessment registration revalidation process
	Economic factors	1.19	Macroeconomic factors
		1.20	Economic and political stability
		1.21	Recession/economic instability
		1.22	Devaluation of money
		1.23	Remittance to home coutnry
		1.24	Changes to renumeration
		1.25	Corruption in everyday life
	Political factors	1.26	Political situation
		1.27	Policy issues
		1.28	Safety for family, self, fleeing violence
		1.29	Bilateral agreements
		1.30	Immigration policies
		1.31	Ease of obtaining right to remain
		1.32	Ease of movement to the UK from EU
		1.33	Ease of movement within the EU
		1.34	Citizenship status
		1.35	UK referendum vote to leave EU
		1.36	Colonial connections
		1.37	Compulsory service in the public sector
	Social factors	1.38	Social conditions
		1.39	Promotes multiculturalism
		1.40	History culture of medical migration
		1.41	Historical ties
		1.42	Xenophobia discrimination
		1.43	Gender equity
		1.44	Unequal opportunities
		1.45	Established networks
2. Meso-level drivers	Training opportunities	2.1	Better training and development opportunities
		2.2	Desire to learn the state of the art in the profession
		2.3	Status of gaining qualifications and training from specific country
		2.4	Opportunity to advance knowledge and education of self
		2.5	Opportunity to advance knowledge, skills of sector country
		2.6	Lack of professional development opportunities
		2.7	Shortage of postgraduate training opportunities
		2.8	Shortage of posts in a particular specialty profession
		2.9	Poor standard of training
	Employment opportunities	2.10	Desire to experience working in a different environment
		2.11	Better working relationships
		2.12	Job satisfaction experience
		2.13	Poor working relationships
		2.14	Pushed desire to leave the NHS
		2.15	Poor intellectual stimulation
		2.16	Healthcare professionals are valued
		2.17	Undervalued professionally
	Career progression opportunities	2.18	Career progression
		2.19	Opportunities to gain clinical experience through short-term employment
		2.20	Opportunity for research
		2.21	Opportunity for networking
		2.22	Lack of promotion
		2.23	Limited career opportunities
		2.24	Negative research environment
		2.25	Healthcare structure, management issues
3. Micro-level drivers	Personal fulfilment	3.1	Better quality of life
		3.2	Desire for life change
		3.3	Adventure
		3.4	Family reasons
		3.5	Better education for children
		3.6	Better climate environment
		3.7	Future hopes and goals
		3.8	Better morale and well-being
		3.9	Personal growth
		3.10	To be competitive enhance CV
		3.11	To provide better patient care
		3.12	Improve languages
		3.13	Humanitarian work
		3.14	Poor work–life balance quality of life
		3.15	Lack of morale
		3.16	Burnout stress
	Financial factors	3.17	Financial gain for self
		3.18	Financial gain for family
		3.19	Scholarship
		3.20	Financial hardship
	Location factors	3.21	Proximity Location of destination country
		3.22	Stepping stone to another destination
		3.23	Prior experience in destination country
		3.24	Common Language
		3.25	Language problems
	Themes and descriptions	Codes	Label
4. Macro-level barriers	Health system factors	4.1	Healthcare system difficult to enter
		4.2	Institutional change
		4.3	Improvements to healthcare system in home country
		4.4	Increased salary
		4.5	Investment in working and living conditions
		4.6	Creating job opportunities
		4.7	Workforce demand
		4.8	Healthcare workers are valued in home country
		4.9	Poor salaries
		4.10	Poor working conditions
		4.11	Limited employment opportunities
		4.12	Decrease in workforce demand
		4.13	Underemployment skills loss
		4.14	Limitations on recruitment
		4.15	Limiting the number of students seeking medical education training
		4.16	Bureaucratic process
	Economic factors	4.17	Economic crisis
		4.18	Financial loss for country
		4.19	Financial support
	Political factors	4.20	Political situation
		4.21	Stricter immigration policies
		4.22	Policy changes
		4.23	Bilateral agreement
		4.24	Lack of citizenship
		4.25	Obtaining residence permit
		4.26	Process of gaining registration
		4.27	Bonded to work in home country
		4.28	Long emigration process
		4.29	UK decision to leave the EU
	Social factors	4.30	Xenophobia discrimination
		4.31	Cultural factors
		4.32	Gender
		4.33	Religious factors
5. Meso-level barriers	Training factors	5.1	More domestic training opportunities
		5.2	Investment in language training
		5.3	Limited training opportunities
		5.4	Restructure of training process
		5.5	Difficult to get a specialist training post
		5.6	Expensive examinations
		5.7	Qualifications undervalued
		5.8	Training deanery
		5.9	Cost of training relocation
	Employment factors	5.10	Improving career opportunities
		5.11	Provision of professional development opportunities
		5.12	Positive working relationships
		5.13	Measures to prevent burnout
		5.14	Negative job security/short-term job contract
		5.15	Lack of recognition of qualifications or experience
		5.16	Negative induction scheme
6. Micro-level barriers	Personal factors	6.1	Family ties
		6.2	Better quality of life in home country
		6.3	Concerns about starting a new life experience
		6.4	Loyalty to profession in home country
		6.5	Homesickness
		6.6	Stress and or isolation
		6.7	Long-term settlement plan
	Employment factors	6.8	Concerns about a new working environment
		6.9	Concerns regarding Appraisal revalidation certification
		6.10	Good job in home country
		6.11	Higher workplace satisfaction
		6.12	Lack of work experience
		6.13	Negative experiences
		6.14	Lack of references from destination country
	Financial factors	6.15	Financial loss for self family
		6.16	Government scholarship
		6.17	Potential loss of employment benefits
	Social factors	6.18	Social status
		6.19	Lack of support
		6.20	Lack of overseas network
	Location factors	6.21	Limited knowledge of destination country
		6.22	Language difficulties

## Appendix C

### Characteristics of included studies

**Table:** 

	Author	Year	Aim of study	Methods	Country	DRIVER CODES	BARRIER CODES
1	Adebayo [51]	2021	To assess the emigration intentions of doctors undergoing residency training in a tertiary healthcare centre in Nigeria and the factors that influence these intentions	Mixed Methods	Africa/Asia	3.1, 1.6, 3.17, 3.4, 1.13, 2.4, 2.1	
2	Bailey [59]	2012	To explore the factors influencing the career plans of medical students and recent graduates with regard to four policy-relevant aspects	Qualitative	Africa/Asia	1.12, 1.16, 1.3, 2.10, 2.12, 2.17, 2.5, 2.7, 3.17, 3.18	4.21, 4.22, 6.4, 6.20
3	Bezuidenhout [30]	2009	To investigate the profile of South African qualified physicians who emigrated from South Africa	Quantitative	Africa/Asia	1.5, 3.17	4.10, 4.13,
4	Labonté [61]	2015	To better understand the drivers of skilled health worker migration, its consequences, and the strategies countries have employed to mitigate negative impacts	Mixed Methods	Africa/Asia	1.19, 1.21, 1.25, 1.1, 1.13, 1.14, 1.15, 1.16, 1.5, 1.6, 1.28, 2.18, 2.17, 2.6, 3.17, 3.20, 3.1, 3.16, 3.4	4.17, 4.12, 4.14, 4.3, 4.5, 4.23, 4.26, 4.31, 6.1, 6.2
5	Gureje [29]	2009	To understand the brain drain of health professionals	Mixed methods	Australia	1.1, 1.14, 1.15, 1.5, 1.6, 1.28, 2.24, 2.25, 2.12, 2.13, 2.1, 2.4, 2.9, 3.17, 3.1, 3.5	
6	McDermott [44]	2015	To explore the increasing numbers of emergency medicine (EM) registrars that obtained their primary medical degree from UK or Irish universities, who work in emergency departments (ED) throughout Australia and New Zealand	Quantitative	Australia	1.21, 1.16, 1.6, 2.25, 2.14, 3.1	4.21
7	Mara [57]	2020	To analyse the recent trends in the mobility of health professionals in Europe	Mixed Method	Europe	3.17	
8	Ramos [47]	2017	To determine the prevalence of migration intentions among Portuguese junior doctors and to identify the most important drivers of career choice for those who are considering migrating in the near future	Quantitative	Europe	1.16, 1.5, 2.20, 3.17	
9	Schumann [60]	2019	To explore the driving forces in a group of Egyptian physicians and final-years medical students preparing to migrate to Germany	Qualitative	Europe	1.13, 1.18, 1.3, 1.5, 1.40, 1.45, 2.25, 2.1, 2.7,	
10	Ognyanovaa [40]	2012	To shed light on the changes in the scale of movement, trends and directions of flows of health professionals pre and post 2004 and 2007 EU enlargements	Quantitative	Europe	1.1, 1.6, 1.33, 2.1, 3.17, 3.21	4.4, 4.5, 4.21,
11	Wismar [39]	2011	To enhance knowledge on the nature and extent of health professional mobility in the EU, assess its impact on country health systems and outline some major policy strategies to address mobility	Quantitative	Europe	1.20, 1.21, 1.23, 1.25, 1.1, 1.11, 1.12, 1.14, 1.15, 1.16, 1.2, 1.3, 1.4, 1.6, 1.8, 1.9, 1.26, 1.27, 1.31, 1.32, 1.33, 1.38, 1.44, 2.18, 2.19, 2.20, 2.21, 2.24, 2.25, 2.10, 2.12, 2.16, 2.17, 2.1, 2.2, 2.3, 2.4, 2.6, 2.7, 2.8, 2.9, 3.17, 3.21, 3.22, 3.23, 3.24, 3.25, 3.1, 3.11, 3.12, 3.15, 3.2, 3.3, 3.4, 3.5	4.11, 4.21, 4.26, 6.21, 6.22, 6.1, 5.14, 5.15, 5.3
12	Buchan [42]	2014	To assess the scale of mobility of health professionals from the new to the old EU Member States before and after the 2004 and 2007 EU enlargements	Quantitative	Europe	1.20, 1.21, 1.23, 1.25, 1.1, 1.11, 1.12, 1.13, 1.15, 1.16, 1.3, 1.6, 1.8, 1.26, 1.32, 1.38, 1.45, 2.18, 2.19, 2.20, 2.21, 2.25, 2.10, 2.13, 2.17, 2.1, 2.2, 2.3, 2.6, 2.7, 2.8, 3.17, 3.22, 3.24, 3.1, 3.11, 3.12, 3.14, 3.2, 3.3, 3.4, 3.5	4.1, 4.11, 4.21, 4.31, 6.22, 6.1, 6.3, 6.4, 6.18, 5.15, 5.3
13	Bidwell [49]	2012	To explore the extent of increased dependence on international medical migration which has both national and international policy implications (2000–2010)	Quantitative	Ireland	1.2, 2.18	4.9, 4.26, 5.14
14	Humphries [74]	2013	To provide insight into the experiences of non-EU migrant doctors in the Irish health workforce	Qualitative	Ireland	1.12, 1.16, 2.1, 2.6	
15	Humphries [52]	2021	To ascertain whether (and how) the COVID‑19 pandemic might disrupt or reinforce existing patterns of doctor emigration	Qualitative	Ireland	2.1, 1.46	
16	Wójcicka [28]	2009	To provide a national profile of migration of health professionals in Ireland	Qualitative	Ireland	1.20, 1.21, 1.10, 1.11, 1.14, 1.16, 1.18, 1.3, 1.4, 1.5, 1.32, 1.40, 1.45, 2.1, 2.3, 2.7, 3.17, 3.21, 3.1	4.11, 4.9
17	Begum [58]	2019	To examine how many senior scientists and clinicians were from other countries, particularly from Europe, in two time periods	Quantitative	UK	1.8, 1.30	4.20, 4.21,
18	Blacklock [43]	2012	To investigate the effect of UK policy on medical migration	Quantitative	UK	1.1, 1.30,	4.23
19	Bornat [25]	2011	To examine the push/pull factors and oral histories of overseas trained doctors from South Asia who have entered the UK workforce in geriatric medicine	Qualitative	UK	1.5, 2.3	
20	Crossland [56]	2021	To report the numbers of consultant congenital cardiac surgeons and cardiologists who have joined and left UK practice over the last 10 years and explore the reasons for leaving	Quantitative	UK	3.9, 3.17, 2.14, 3.14, 2.1, 2.20, 2.8	
21	Davda [20]	2018	To examine the migration motives, the barriers to and facilitators of integration of international dental graduates, compared with nurses and doctors in the United Kingdom	Literature review	UK	1.21, 1.22, 1.24, 1.25, 1.1, 1.11, 1.12, 1.13, 1.15, 1.16, 1.5, 1.7, 1.29, 1.30, 1.45, 2.19, 2.22, 2.10, 2.1, 2.7, 3.1, 3.18, 3.1, 3.2, 3.3, 3.5	
22	Gauld [36]	2015	To examine why these doctors go to New Zealand and do not stay for long	Quantitative	UK	1.5, 1.6, 2.14, 2.1, 3.17, 3.1	6.1
23	George [55]	2017	To examine the salaries of selected HRH in India and four popular destination countries (United States of America, United Kingdom, Canada and the United Arab Emirates) while accounting for the in-country cost of living	Quantitative	UK	3.17	
24	Herfs [26]	2014	To present data relating to the changes in IMG migration in the UK since the extension of the European Union in May 2004. In addition, data are presented on IMG migration in the Netherlands. These migration flows show that migration patterns differ strongly within these two EU-countries	Quantitative	UK	1.18, 1.5, 1.40, 2.1, 2.4, 3.17, 3.24	
25	Hosni [50]	2017	To find out if doctors leaving the UK at the end of the 2 year "International Doctors Training Programme of Obstetrics and Gynaecology" feel that they achieved what they expected to achieve, what went well and what did not go well	Quantitative	UK	2.19, 2.10, 2.1, 3.2	
26	Iacobucci [14]	2017	To explore if EU doctors are considering leaving UK	Quantitative	UK	1.30, 1.35, 1.42,	
27	Lambert [34]	2017	To report the changes to UK medicine which doctors who have emigrated tell us would increase their likelihood of returning to a career in UK medicine	Quantitative	UK	1.14, 1.15, 1.16, 1.18, 2.20, 2.25, 2.17, 3.17, 3.1, 3.15, 3.4, 3.6	4.21, 5.14
28	Legido-Quigley [27]	2015	To describe the experiences of doctors who decide to move to the UK from other EU member states, exploring their motivations for moving and their experiences of registering and working in the UK	Qualitative	UK	1.13, 1.5, 2.1, 2.4, 3.17, 3.24, 3.12, 3.9	4.1, 4.12, 4.26, 6.22, 5.16
29	Milner [71]	2021	To assess how Brexit relates to doctors’ migration intentions in relation to their feelings that Brexit has impacted their professional life, national identity, and demographic factors	Quantitative	UK	1.35	
30	Milner [75]	2021	To provide a detailed examination of European doctors’ feelings towards Brexit, their intentions to leave the UK, and factors that may contribute to their potential decisions to migrate	Quantitative	UK	1.35	
31	Quantin [37]	2012	To analyse the migration of doctors between the UK and France, in an attempt to identify the reasons for these migrations	Quantitative	UK	1.16, 1.6, 1.7, 2.18, 2.1, 3.17, 3.2, 3.4, 3.6	
32	Sharma [33]	2012	To investigate factors which influenced UK-trained doctors to emigrate to New Zealand and factors which might encourage them to return	Quantitative	UK	1.14, 1.5, 2.12, 2.14, 3.23, 3.1, 3.2, 3.4	4.3,
33	Smith [62]	2012	To conduct an exploratory study to learn about the experiences of GPs who have undertaken international work	Quantitative	UK		6.9, 6.15, 6.17, 6.1, 6.19, 5.14, 5.8
34	Smith [32]	2018	To explore the reasons that doctors choose to leave UK medicine after their foundation year 2 posts	Quantitative	UK	1.19, 1.13, 1.16, 1.6, 1.26, 1.30, 1.32, 2.19, 2.11, 2.13, 2.16, 2.17, 2.1, 2.6, 2.9, 3.1, 3.14,	4.29,
35	Torjesen [54]	2017	To examine the affect of Brexit on EEA doctors intent to continue working in the UK	Quantitative	UK	1.35, 2.17, 3.15	
36	Van der Pol [31]	2019	To examine the association between risk attitudes and the migration of UK GPs to Australia	Quantitative	UK	1.5, 2.14	
37	Khan [48]	2015	To review and inform the relevant authorities about the barriers faced by IMGs in training and career progression in the UK health service	Literature Review	UK	1.28, 2.19, 2.20, 2.10, 2.1, 2.4, 3.17, 3.1	4.1, 4.26, 6.8, 6.6, 6.19
38	Lambert [34]	2017	To report the reasons why doctors are considering leaving medicine or the UK	Quantitative	UK	1.16, 1.5, 1.6, 2.10, 2.14, 2.1, 3.17, 3.1, 3.11, 3.14, 3.4	
39	BMA [38]	2010	To provide information on the careers of doctors, and particularly to: –– identify doctors who leave medicine as a career, or who choose to work in another country and to assess the factors which influence it	Mixed Methods	UK	1.6, 2.19, 2.10, 2.14, 3.1, 3.13, 3.2	
40	Young [45]	2010	To scope the main issues and identify gaps in knowledge around two key aspects of health professional mobility/migration – within- UK mobility (i.e. movement between England, Scotland, Wales and Northern Ireland) and mobility to and from the different UK countries and Europe	Mixed Methods	UK	1.23, 1.11, 1.12, 1.15, 1.16, 1.26, 1.32, 1.40, 1.45, 2.18, 2.19, 2.20, 2.21, 2.10, 2.1, 2.2, 2.3, 2.7, 2.8, 3.18, 3.24, 3.1, 3.12, 3.2, 3.3, 3.4, 3.7	6.8, 6.22

## Data Availability

Not applicable.
